# Combining Fixed-Weight ArcFace Loss and Vision Transformer for Facial Expression Recognition

**DOI:** 10.3390/s25237166

**Published:** 2025-11-24

**Authors:** Yunhao Xu, Xinran Duan, Peihao Fan, Zengshun Zhao, Xiaoyu Guo

**Affiliations:** 1 College of Electronic and Information Engineering, Shandong University of Science and Technology, Qingdao 266590, China; 2 China United Network Communications Co., Ltd. (CUCC), Qingdao Branch, Qingdao 266000, China

**Keywords:** deep learning, facial expression recognition, Vision Transformer, ArcFace loss

## Abstract

In recent years, deep learning has demonstrated remarkable capability in the broad domain of feature learning. Although the facial expression recognition task has been extensively studied, it still faces many challenges such as significant variations within the same category of expressions. ArcFace loss is a widely adopted function designed to enhance inter-class separability and improve recognition performance. However, it does not explicitly constrain the angular distribution between class centers. Therefore, this study introduces a weight-constrained ArcFace loss and integrates it into the Vision Transformer (ViT) framework. This approach not only alleviates implicit biases induced by imbalanced data distributions but also significantly reduces computational overhead by stabilizing weight optimization. In the experiments, the authors evaluated the proposed approach against standard ArcFace loss, classical loss functions, and various network structures on the RAF-DB and FER2013 datasets. Comprehensive experimental results demonstrate that the proposed approach not only improves recognition accuracy but also achieves higher computational efficiency.

## 1. Introduction

Facial expressions constitute one of the most powerful and natural signaling systems for conveying human emotions and intentions. Facial expression analysis has found broad applications in affective robotics, medical rehabilitation, driver fatigue monitoring, and human–computer interaction [1,2]. As early as 1971, Ekman and his colleagues [3] identified six basic facial expressions—anger, disgust, fear, happiness, sadness, and surprise—along with the neutral state. However, in real-world scenarios, facial expressions exhibit considerable variability stemming from differences in age, gender, cultural background, and individual expressiveness. Additionally, challenges such as pose variations, illumination changes, and partial occlusions are common in unconstrained recording environments. The complex nonlinear interactions among these factors and facial features result in high intra-class variance and low inter-class discriminability. In other words, features of similar expressions are widely dispersed, while those of different expressions often exhibit strong similarity [4,5,6,7]. Consequently, the accuracy of facial expression recognition remains relatively limited, and the field continues to face significant technical challenges [8].

In facial expression recognition tasks, traditional convolutional neural networks employ Softmax loss functions [9,10,11,12] to enhance inter-class separability. However, this approach fails to effectively account for intra-class feature variability. To address this limitation, various margin-based and metric-learning loss functions have been proposed [13]. Notably, the island loss function is inspired by the center loss used in face recognition tasks [14]. By introducing an additional penalty term to minimize intra-class distances, island loss further enhances inter-class separation by additionally reducing cosine distances between class centers. This dual mechanism not only strengthens intra-class compactness but also enlarges the spacing between clustering centers [15].

To address the challenges of large intra-class variations and small inter-class separations in face recognition tasks [16,17,18], Deng et al. [19] proposed the ArcFace loss, which introduces an additive angular margin into the Softmax framework to directly maximize the classification margin in angular space. Building on this principle, margin-based angular losses, such as ArcFace, have been widely adopted to enhance inter-class separability by enforcing additive angular margins within the normalized feature space. By expanding the angular decision boundaries, ArcFace enhances both intra-class compactness and inter-class discrimination, particularly in large-scale face recognition (FR) datasets where sufficient training samples facilitate stable optimization. However, its effectiveness hinges on the implicit assumption that class centers (i.e., classifier weights) naturally disperse into a well-separated configuration during training. This assumption, however, does not hold in facial expression recognition tasks, where datasets are typically smaller, suffer from severe class imbalance, and contain highly correlated expression categories [20,21,22,23,24]. Under these conditions, ArcFace tends to yield non-uniform class-center distributions, unstable angular boundaries, and biased predictions that favor majority classes [25].

Recent advances in hyperspherical feature learning [26,27,28] have shown that explicitly constraining the angular distribution of class centers is essential for achieving uniform separation and stable convergence. However, standard angular-margin losses—including ArcFace, AM-Softmax, and CosFace—focus solely on enforcing margins between features and their corresponding class weights, leaving the global angular relationships among different class centers largely unconstrained. This structural limitation becomes particularly problematic in facial expression recognition (FER), where minority expression categories (e.g., disgust, fear) lack sufficient samples to adequately separate their class centers from those of dominant classes. Existing approaches such as EvenFace, UniformFace, and DuaFace [26,27,28] attempt to promote class-center uniformity; however, they often rely on complex architectural modifications and incur high computational costs and have been rarely applied to FER.

These limitations reveal a clear methodological gap—specifically, the absence of explicit angular constraints among class centers when ArcFace-like losses are applied to FER [29]. To bridge this gap, we propose a fixed-weight ArcFace loss that enforces uniform angular separation by maximizing pairwise angular distances among class centers. This constraint enhances angular coverage and inter-class balance, thereby improving the discriminability of minority expressions without altering the feature extractor.

Owing to its self-attention mechanism, the Vision Transformer (ViT) can model long-range dependencies among spatially distant facial regions, making it particularly suitable for capturing structural details crucial for FER. However, the integration of ViT with angular-margin losses has not been thoroughly investigated. It also remains unclear whether imposing explicit angular constraints on class-center weights can further enhance the discriminative capability of Transformer-based classifiers. To more effectively capture subtle expression variations across facial regions, we integrate the proposed loss function into the ViT framework, which enhances inter-class separability and improves recognition of minority expressions [30,31]. Furthermore, fixing the classifier weights removes backpropagation through the final classification layer, thereby improving training stability and reducing computational overhead [32].

Comprehensive experiments on RAF-DB and FER2013 validate the effectiveness of the proposed framework. Our method consistently outperforms Softmax, Island loss, and standard ArcFace across multiple architectures, with particularly notable gains for minority and visually similar expressions. These results confirm the robustness and practical utility of explicitly enforcing class-center angular constraints in Transformer-based FER systems.

The authors’ major contributions are as follows:

- Exploring and analyzing the loss function in the face recognition task in depth, through further analysis of ArcFace loss, the authors add a new loss term with a constrained weight vector to the fully connected layer on top of the ArcFace loss. This design enforces a uniform angular distribution of class centers, enhancing intra-class compactness and inter-class separability. Consequently, it improves recognition of minority expression categories and mitigates the adverse impact of data imbalance on classification performance.
- The model features a lightweight and efficient training design. Under the constraint on the weights, the classification layer no longer needs to back-propagate to update the weights, which significantly reduces the number of parameters and gradient calculation overhead. This leads to a substantial reduction in training time and computational cost.
- Under the FER2013 [33] and RAF-DB [34] facial expression databases, the authors conducted experiments using Vision Transformers and traditional convolutional networks to perform feature extraction tasks. The authors systematically compared the proposed method with traditional loss functions and the standard ArcFace loss function, yielding consistent and reliable improvements.

## 2. Proposed Approach

To facilitate understanding of the mathematical formulations and derivations presented in this section, we firstly define the key symbols and their meanings in a nomenclature table. Readers may refer to this table to familiarize themselves with the notations. This helps improve the readability and clarity of the subsequent method descriptions. Table 1 summarizes the symbols and definitions related to the angular constraints and loss functions used in facial expression recognition.

### 2.1. Review of ArcFace Loss

In the field of facial recognition, due to the limitations of the Softmax loss function, many optimized loss functions have been proposed. For example, the LargeMargin (L-softmax) loss function [35], AngularMargin (AM-softmax) loss function [36], SphereFace loss function [37], and CosFace loss function [38] are all representative improvement schemes.

To further improve recognition accuracy and training stability, Deng et al. [19] from Imperial College London introduced the ArcFace loss in 2018. Its core principle is to calculate the cosine similarity between the normalized deep feature vectors extracted by the DCNN [39] and the normalized class-center weights of the final fully connected layer.

The most widely used classification loss function Softmax can be expressed as follows [35]:

(1) $$L_{1}\;=\;-\frac{1}{N}\sum_{i=1}^{N}\log\frac{e^{W_{y_{i}}^{T}x_{i}+by_{i}}}{\sum_{j=1}^{n}e^{W_{j}^{T}x_{i}+b_{j}}}\;\;\;\;\;$$

where $x_{i}$ represents the corresponding feature vector of the i sample and belongs to the $y_{i}$ category. $W_{j}\;$ is the j column of the weight matrix W, and $b_{j}$ is the offset term. The batch size of the input image is N, and the total number of categories in the training set is n. The limitations of the Softmax loss function lie in its failure to explicitly optimize feature embeddings; thus it is unable to effectively improve the similarity between intra-class samples and the diversity of inter-class samples. Therefore, when applied to facial expression recognition testing scenarios with significant changes in appearance, this function may experience performance shortcomings.

For simplicity, we will set the offset term $b_{j}$ to 0, and then convert the logits item to [19]:

(2) $$W_{j}^{T}x_{i}\;=\;\left\|W_{j}\right\|\left\|x_{i}\right\|\cos\theta_{j}$$

where $\theta_{j}$ is the angle between the weight $W_{j}$ and the feature $x_{i}$. First, the norm of the individual weight $W_{j}$ is determined by $L_{2}$ normalization, and then the embedded feature $x_{i}$ is normalized and rescaled to s. The normalization step of features and weights makes the prediction only depend on the angle between features and weights. Therefore, the learned embedded features are distributed on a hypersphere with a radius of 1 [19]:

(3) $$L_{2}\;=\;-\frac{1}{N}\sum_{i=1}^{N}\log\frac{e^{s\cos\theta_{yi}}}{e^{s\cos\theta_{yi}}+\sum_{j=1,j\neq yi}^{n}e^{s\cos\theta_{j}}}$$

An additional angular margin penalty term m is added between $x_{i\;}$ and $W_{yi}$ to simultaneously enhance intra-class compactness and inter-class differences, since the embedded features are distributed around each corresponding feature center on the hypersphere. The additional angular margin penalty term proposed in the ArcFace paper is equal to the geodesic distance margin penalty term in the hypersphere [19]:

(4) $$L_{3}\;=\;-\frac{1}{N}\sum_{i=1}^{N}\log\frac{e^{s\left(\cos\left(\theta_{yi}+m\right)\right)}}{e^{s\left(\cos\left(\theta_{yi}+m\right)\right)}+\sum_{j=1,j\neq yi}^{n}e^{s\cos\theta_{j}}}$$

The first step of the face classifier training process is to normalize the feature vector and weight $W_{j}$, and then the cosine of the angle between them is calculated. Secondly, the inverse cosine is used to obtain the angle between the feature $x_{i}\;$ and the target weight $W_{yi}$, add the angular margin m, and then calculate the $\cos\left(\theta +m\right)$. Finally, all logits are multiplied by the feature scale s and sent to the Softmax function to obtain the probability of each category. Ground Truth and One-Hot Vector are used to calculate cross-entropy loss, that is, the dot product between the DCNN feature and weights of the last fully connected layer equals the cosine distance after the feature and weight are normalized.

### 2.2. Advanced Fixed Weight

**Figure 1:**
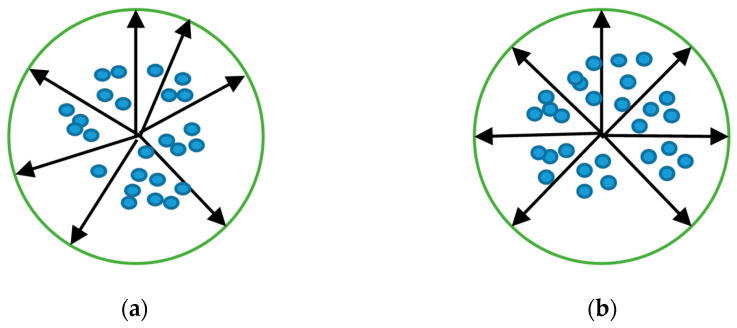
(**a**) Feature distribution trained with the ArcFace loss, where the absence of a global constraint leads to uneven class-center distribution; (**b**) Feature distribution trained with the fixed-weight ArcFace loss, where the explicit angular constraint produces uniformly distributed class centers and features, thereby enhancing the inter-class separability and better accommodating the class-imbalance characteristics of FER.

Although ArcFace enhances inter-class separability by introducing an additive angular margin, its formulation inherently focuses solely on the local relationship between a feature $x_{i}$ and its corresponding class weight $W_{y_{i}}$. Specifically, ArcFace modifies the positive class logit as: $cos(\theta_{yi})\to cos(\theta_{yi}+m),$ and its loss function form is $L_{3}$. While this margin improves the angular separation between each sample and its ground-truth weight vector, ArcFace does not impose any constraint on the angular distribution among the class centers $\left\{W_{j}\right\}$ themselves. As a result, on small and imbalanced FER datasets, the classifier weights often converge into a narrow angular region, because the gradients contributed by minority classes are insufficient to push their class centers apart. The illustrations of the feature distribution map of the standard ArcFace are shown in Figure 1a. Moreover, visually similar expression categories tend to yield overlapping feature directions, resulting in small inter-class angles that ArcFace cannot correct due to its purely sample–center formulation.

Therefore, in response to the above-mentioned issues, the authors explicitly constrain the geometric structure of the classifier by introducing fixed, uniformly distributed weight vectors and an angular diversity regularization term. Specifically, all class weights are initialized as unit-norm vectors uniformly distributed on the hypersphere [40] and remain non-trainable during optimization, preventing gradient imbalance from drifting the classifier geometry. The feature distribution map using the ArcFace loss with fixed weights is shown in Figure 1b. To further enforce global angular separation, we define the regularization term:

(5) $$L_{4}\;=\;\omega \sum_{i\in N}\sum_{\begin{array}{c} j\in N\\ i\neq j \end{array}}\frac{W_{i}^{T}\cdot W_{j}}{{\left\|W_{i}\right\|}_{2}{\left\|W_{j}\right\|}_{2}}=\;\omega \sum_{i\neq j}cos(\theta_{ij})$$

where $\theta_{ij}$ denotes the angle between class centers $W_{i}$ and $W_{j}$. By minimizing $L_{4}$, the pairwise cosine similarity among class centers is reduced, leading to an approximately optimal hyperspherical coding configuration [41]. Unlike the standard ArcFace, which regulates only the local angle between a feature and its corresponding class center, the joint use of fixed weights and the global angular constraint in $L_{4}$ ensures uniformly distributed class centers. This design enhances inter-class separability and mitigates the adverse effects of class imbalance in FER.

To obtain the class-center weights that are uniformly distributed on the hypersphere, each weight vector $W_{i}$ is first randomly initialized and normalized on the unit hypersphere. During iterative optimization, pairwise angular similarities among all class centers are computed, and weights are updated following the gradient of the angular regularization term defined in Equation (5). As a result, this procedure progressively reduces the maximum inter-class similarity, driving the class centers toward a more uniform angular configuration. After convergence, the resulting weight matrix W is fixed and used throughout the entire training process. To balance inter-class separation and intra-class compactness, the weight of the regularization term ω in Equation (5) is set to 1/2 based on the grid search experiments. Specifically, ω values in {0.1, 0.3, 0.5, 0.7, 0.9} were tested on the RAF-DB validation set, and ω=0.5 achieved the best trade-off between inter-class separation and intra-class compactness.

Overall, this strategy provides a stable and reproducible initialization of class centers, enhances global angular separability, and overcomes the limitation of standard ArcFace, which relies solely on implicit margin optimization without explicitly regulating the geometric structure of class-center distributions. The following procedure in Algorithm 1 outlines the method used to compute the category weights for ArcFace loss:
**Algorithm 1:** computing weights for Arcface loss**Input:** learning rate α$,\;\mathrm{the}\;\mathrm{angle}\;\mathrm{of}\;W_{i}$$\;\mathrm{and}\;W_{j}$$\;\mathrm{as}\;A_{ij}$$1.\;\mathrm{Randomly}\;\mathrm{initialize}\;W_{i}$$2.\;\mathrm{Normalize}\;W_{i}$$\;\mathrm{to}\;\left\|W_{i}\right\|=1$**Training step:**$3.\;C=\underset{\begin{array}{c} i,j\in N\\ i\neq j \end{array}}{max}(A_{ij})=\underset{\begin{array}{c} i,j\in N\\ i\neq j \end{array}}{max}\left(\frac{W_{i}^{T}\cdot W_{j}}{{\left\|W_{i}\right\|}_{2}{\left\|W_{j}\right\|}_{2}}\right)$$4.\;\mathrm{if}\;C\in \left(0,\pi \right)$ then$5.\;\mathrm{Calculate}\;\mathrm{the}\;\mathrm{loss}\;L_{4}$ as in Equation (5)$6.\;\mathrm{Update}\;\mathrm{the}\;\mathrm{weight}\;W_{i}-\alpha ∆W_{i}\to W_{i}$$7.\;W_{i}^{t+1}=W_{i}^{t}-\alpha^{t}\cdot \frac{{\partial L}_{4}^{t}}{{\partial w}_{i}^{t}}$8. End while**Output:** $W_{i}\left(i\in N\right)$

In summary, the innovative loss function we proposed in this paper is as follows:

(6) $$L\;=\;L_{3}+\varphi L_{4}=\;-\frac{1}{N}\sum_{i=1}^{N}\log\frac{e^{s\left(\cos\left(\theta_{yi}+m\right)\right)}}{e^{s\left(\cos\left(\theta_{yi}+m\right)\right)}+\sum_{j=1,j\neq yi}^{n}e^{s\cos\theta_{j}}}+\varphi \times \omega \sum_{i\neq j}cos(\theta_{ij})$$

where $L_{3}$ imposes a local angular margin between each feature and its corresponding class center, while $L_{4}$ explicitly regulates the global angular distribution among all class centers [42]. The hyperparameter φ controls the relative weight of the global angular diversity regularization term $L_{4}$ in the overall loss L.

The angular margin m and scaling factor s in Equation (6) are optimized via cross-validation to achieve a balance between discriminative feature learning and stable convergence. Specifically, we evaluate m∈{0.1, 0.2, 0.3, 0.4} and find that m=0.3 achieves the most balanced performance on FER datasets. These datasets typically exhibit imbalanced label distributions and subtle inter-class differences. An overly small margin results in inadequate angular separation, while an excessively large one may destabilize training, particularly for minority classes. The scaling factor s is fixed at 30, following prior angular-margin studies, to maintain consistent feature magnitudes across datasets and to prevent overconfident predictions for majority classes. This configuration keeps logits within a numerically stable range and ensures comparability across experiments. Empirically, the combination of m=0.3 and s=30 offers the most reliable trade-off among recognition performance, optimization stability, and cross-dataset generalization.

### 2.3. Expression Feature Extraction Network

#### 2.3.1. Vision Transformer

The Vision Transformer (ViT) exhibits significant advantages in FER tasks. It effectively addresses the limitations of traditional convolutional networks, such as insufficient global context modeling, limited generalization on small datasets, and sensitivity to occlusion or illumination changes.

The multi-head self-attention (MHSA) mechanism establishes global dependencies among facial regions by capturing coordinated movements across multiple facial components, thereby enhancing the model’s understanding of holistic emotional patterns and improving its robustness. Through Patch Embedding, ViT partitions each facial image into fixed-size patches and linearly projects them into the feature space, which helps retain fine-grained local texture information and strengthens the model’s ability to capture subtle emotional variations. The incorporation of Positional Embedding introduces spatial awareness, allowing the model to retain the relative geometric configuration of key facial regions—such as the eyes, mouth corners, and eyebrows—during global context modeling. This design further supports the joint identification of facial action units (AUs) [43]. Furthermore, the global class token aggregates comprehensive semantic information from the entire face to generate a discriminative expression embedding, which serves as a high-quality input for angular-margin-based loss functions such as ArcFace. This synergistic integration of these mechanisms effectively improves inter-class separability and boosts overall recognition accuracy in FER tasks [44].

**Figure 2:**
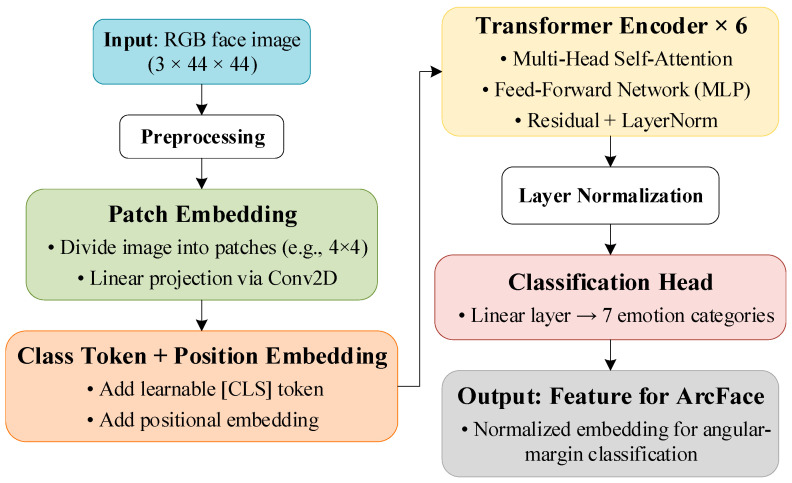
Flowchart of the overall structure of Vision Transformer in facial expression recognition.

To address the limited sample size and low spatial resolution that characterize FER datasets, we employ a lightweight, custom-designed Vision Transformer instead of the standard ViT-Base or ViT-Small architectures. Specifically, the proposed model divides each input image into compact 4×4 patches and employs six Transformer encoder layers to effectively capture the global structural dependencies. This configuration substantially reduces both parameter count and computational overhead, enabling stable training on 44×44 facial images. It also helps mitigate overfitting under data-constrained conditions. The overall ViT architecture adopted in this study is illustrated in Figure 2. The custom-designed lightweight ViT improves training efficiency while preserving strong representational capacity and global modeling ability, thereby producing high-quality embeddings for the subsequent fixed-weight ArcFace classifier.

#### 2.3.2. Lightweight Convolutional Neural Network

To ensure consistency with existing FER studies and evaluate the robustness of the proposed fixed-weight ArcFace loss across different network architectures, we additionally incorporate CNN-based models (ResNet18 and VGG19) into our experiments. These two CNNs provide complementary characteristics: ResNet18 offers a lightweight residual design with strong generalization capability, while VGG19 delivers deeper convolutional feature representations. Both models are among the most widely used and representative baselines in FER [35,36,37,38]. They are particularly effective on medium- and small-scale datasets, such as FER2013 and RAF-DB, where convolutional inductive biases ensure stable and reliable performance.

Notably, CNNs and Transformers model visual information in fundamentally different ways: CNNs rely on local convolutional priors, whereas ViTs capture global dependencies through self-attention mechanisms. Performance comparisons across both paradigms demonstrate that the proposed angular-constrained loss is architecture-agnostic, thereby enhancing its generality and theoretical significance. Hence, incorporating CNN-based baselines aligns with standard FER benchmarking practices and provides essential evidence for the universality and robustness of the proposed loss function.

### 2.4. Proposed Model Based on Advanced Fixed-Weight ArcFace Loss and Vision Transformer

**Figure 3:**
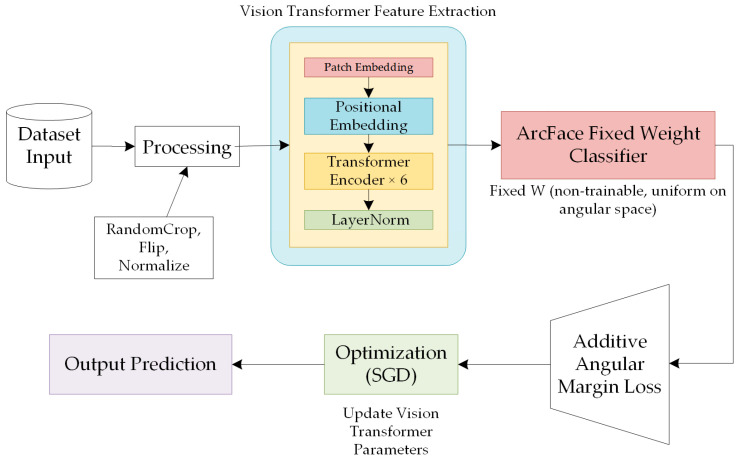
Overall structure diagram of the proposed method.

In this study, the Vision Transformer is integrated with the proposed fixed-weight ArcFace loss to perform facial expression recognition. The overall architecture of the proposed framework is illustrated in Figure 3. The proposed framework comprises four main components: a data preprocessing module, a feature extraction network, a fixed-weight classification layer, and an angular-space loss function.

After the input facial image is processed by the Vision Transformer, its output features are fed into a linear mapping layer (classification head) to generate the final feature embedding. This embedding is then fed into the fixed-weight ArcFace classifier. The classifier’s weight vectors are fixed as uniformly distributed unit vectors on the angular hypersphere and remain non-trainable throughout optimization. This design explicitly constrains the feature distribution within the angular space. By incorporating an angular margin (m) and a scaling factor (s) between the features and the fixed weights, the model effectively enhances inter-class separability while promoting intra-class compactness.

Overall, the improved ArcFace loss is employed as the objective function, replacing the traditional Euclidean distance with angular distance to enhance the discriminative power of the learned features. The model is trained using the SGD optimizer, during which only the parameters of the ViT-based feature extraction network are updated, while the fixed classifier weights remain frozen throughout training. Benefiting from this architectural design, the proposed method achieves high discriminability and robustness, even under challenging conditions such as limited training data, complex illumination variations, and high inter-class similarity in facial expressions.

## 3. Experiment

### 3.1. Preprocessing

**Figure 4:**
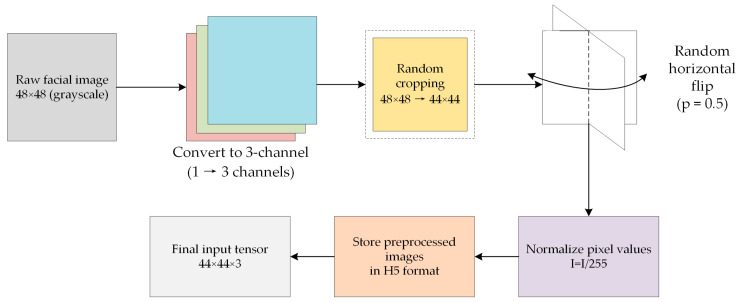
Preprocessing pipeline for FER datasets.

Owing to the limited number of samples in FER datasets, we adopt a standardized preprocessing pipeline to augment data diversity and ensure compatibility with the ViT backbone’s input requirements. The FER2013 dataset provides 48×48 grayscale images, which are firstly uniformly duplicated across three channels to generate pseudo-RGB inputs compatible with both ViT and CNN architectures. To introduce spatial augmentations and improve generalization, each 48×48 image is randomly cropped to 44×44. This resolution also allows the image to be evenly divided into ViT patches without requiring padding. Subsequently, random horizontal flipping (probability = 0.5) is applied, and pixel intensities are normalized to the [0, 1] range by dividing by 255. For consistency, an identical preprocessing strategy is applied to the RAF-DB dataset. Finally, all preprocessed images are stored in H5 format for faster data loading and reduced I/O overhead during training. The overall preprocessing workflow for the facial expression recognition datasets is illustrated in Figure 4.

### 3.2. Experimental Datasets

1. FER2013 Dataset [33]

The dataset comprises a total of 35,886 facial expression images, which are divided into three subsets: 28,708 images for training, 3589 for the public test set, and 3589 for the private test set. Each image is a grayscale facial image with a fixed spatial resolution of 48 × 48 pixels. The dataset includes seven basic facial expression categories, including anger (3995 samples), disgust (436 samples), fear (4097 samples), happiness (7215 samples), sadness (4830 samples), surprise (3171 samples), and neutral (4965 samples).

2. RAF-DB Dataset [34]

The RAF-DB dataset contains a total of 15,339 facial expression images, including 12,271 images for training and 3068 images for testing. The training set comprises seven basic emotion categories, including anger (705 samples), disgust (717 samples), fear (281 samples), happiness (4772 samples), sadness (1982 samples), surprise (1290 samples), and neutral (2524 samples). Each image in the dataset is annotated with a single dominant expression label and collected from diverse real-world conditions, resulting in significant variations in illumination, pose, and occlusion.

### 3.3. Implementation Details

For each benchmark dataset, the Transformer, ResNet18, and VGG19 architectures are employed as backbone networks for feature extraction. The model is optimized with the stochastic gradient descent (SGD) optimizer with a momentum of 0.9 and a weight decay of $5\times 10^{-4}$, applied exclusively to the ViT feature extractor, as the classifier weights are fixed throughout training. Gradient clipping with a threshold of 0.1 is employed to mitigate potential gradient explosion during backpropagation. The initial learning rate is set to 0.01 and kept constant for the first 40 epochs to facilitate stable early-stage optimization. After this initial stage, a piecewise learning-rate decay schedule is adopted, where the learning rate is reduced by a factor of 0.9 every five epochs until training concludes at epoch 200. This schedule enables efficient learning during the early phase while promoting stable and smooth convergence in later training. The batch sizes are configured as 64 for training and 8 for testing. The fixed weight parameter is set to ω = 1/2. For the ArcFace loss, the scaling factor is set to s = 30, and for the RAF-DB dataset, the angle margin m is set to 0.3.

To ensure fair and reproducible evaluation, all experiments are conducted based on the official training and testing splits of RAF-DB and FER2013. During inference, a ten-crop testing strategy is adopted to enhance the robustness of predictions. Each 48×48 test image is cropped into ten 44×44 patches: one center crop and four corner crops (top-left, top-right, bottom-left, and bottom-right). Their corresponding horizontal flips are also included. For each crop, the Vision Transformer extracts a feature vector via its [CLS] token embedding. The ten feature vectors are averaged to obtain a robust aggregated representation of the input image. This averaged feature is subsequently fed into the fixed-weight ArcFace classifier, and the class with the highest cosine similarity score is selected as the final prediction. The batch size is set to 64 during training to fully exploit GPU parallelism, thereby improving computational efficiency and accelerating convergence without compromising stability. In contrast, during inference, each test sample is processed using the ten-crop testing strategy, which produces ten augmented views per image and significantly increases memory usage during forward propagation. To ensure stable and efficient inference within GPU memory limits, the testing batch size is therefore reduced to 8.

Performance is comprehensively evaluated using overall accuracy, per-class accuracy, and the confusion matrix to assess both global and minority class performance. All experiments are repeated three times under identical configurations, and the mean results are reported to ensure statistical reliability.

### 3.4. Experience Results

To evaluate the individual contribution of the proposed loss function, we conduct a systematic ablation study in which the baseline Softmax loss is progressively replaced with the Island loss, ArcFace loss, and finally the proposed angular-constrained fixed-weight loss across the Transformer, ResNet18, and VGG19 architectures. The comparison results are shown in the following Table 2, Table 3 and Table 4.

**Figure 5:**
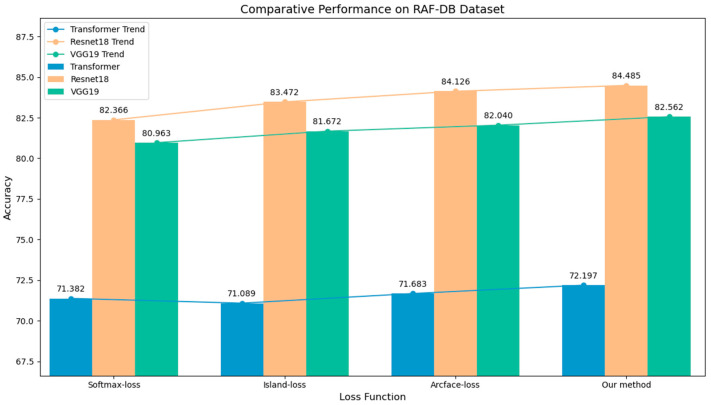
Performance comparison of different loss functions on RAF-DB dataset across Transformer, ResNet18, and VGG19 models.

**Figure 6:**
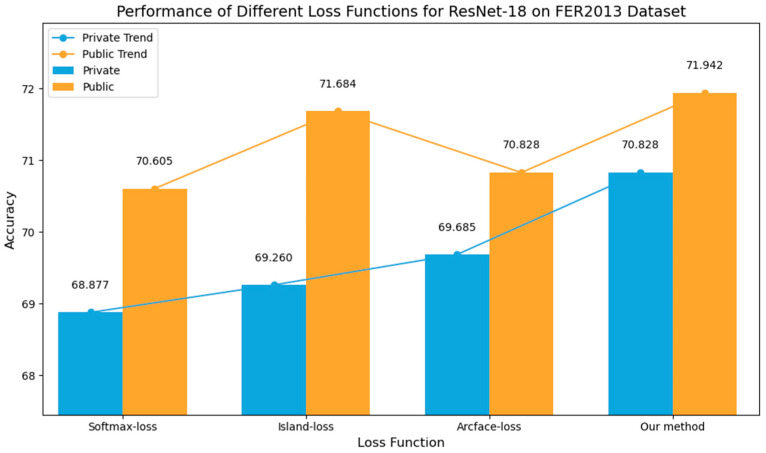
Comparative bar chart of ResNet18 performance on the FER2013 dataset.

On the RAF-DB dataset, the proposed method achieves accuracies of 72.197%, 84.485%, and 82.562% on ViT, ResNet18, and VGG19, respectively, outperforming the standard ArcFace baseline by 0.514%, 0.359%, and 0.522%. On the FER2013 dataset, the proposed method attains accuracies of 70.828% on the Private test set and 71.942% on the Public test set, outperforming the ArcFace loss by 1.143% and 1.114%, respectively. Compared with the other two loss functions, the proposed method achieves a more significant improvement in recognition accuracy. These stepwise improvements confirm that explicitly constraining the angular distribution of features and enforcing dispersion among class-wise weight vectors provide discriminative benefits beyond traditional margin-based losses. The grouped bar charts in Figure 5 and Figure 6 can visually present the performance comparison of the experimental results.

In addition to accuracy, Table 4 reports a reduced computational cost, where the proposed fixed-weight strategy decreases the average training time per epoch on the Transformer to 36.50 s, achieving improvements of 0.34 and 3.12 s over ArcFace and Island loss, respectively. This can be attributed to the core mechanism of the ArcFace loss function—directly optimizing the angular distance between classes without relying on the complex transformations of traditional losses. Moreover, the strategy of fixing the weights in the fully connected layer enhances the training efficiency of ArcFace.

Overall, the ablation results provide compelling evidence that the gains in accuracy, robustness, and efficiency originate primarily from the proposed angular-distribution constraint and fixed-weight classifier design, rather than from any architectural complexity or auxiliary training heuristics.

**Figure 7:**
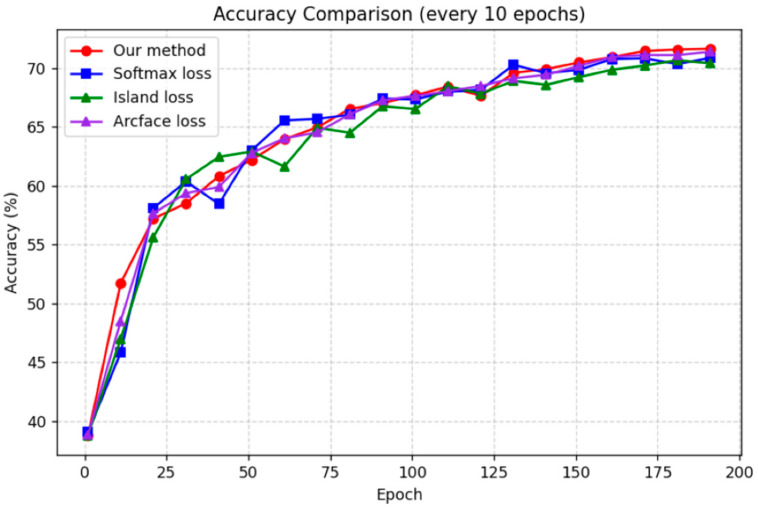
The overall accuracy trends of the four loss functions applied to the transformer model on the RAF-DB dataset.

**Figure 8:**
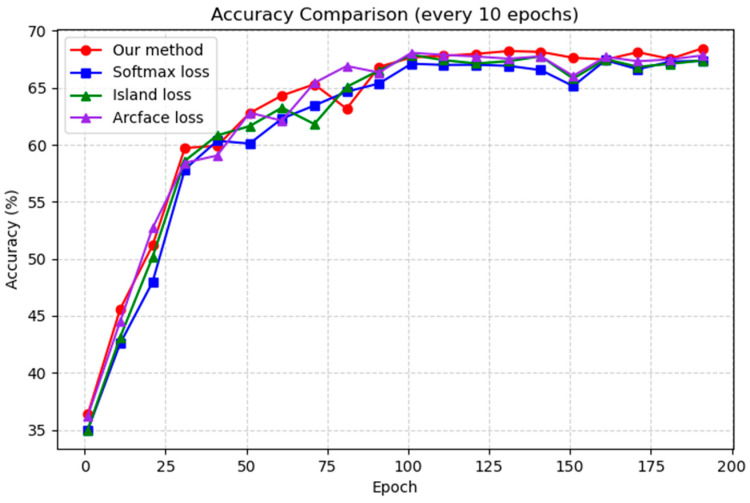
The overall accuracy trends of the four loss functions applied to the transformer model on the FER2013 dataset.

Figure 7 and Figure 8, respectively, present the accuracy convergence curves of the four loss functions on the RAF-DB and FER2013 datasets. During the training process of the Transformer model, the accuracy curves of all four loss functions rapidly converge within the first 50 epochs, indicating that the model achieves effective feature learning in the early stage. In the mid-training phase, the curves continue to rise gradually, but the performance gaps remained small, suggesting similar behavior among the loss functions during this period. After around 140 epochs, however, the proposed approach clearly outperformed the others, achieving both higher final accuracy and faster convergence.

This superior performance validates the effectiveness of the proposed approach through explicit regularization of feature distribution and enforcement of class weight dispersion. In contrast, Softmax loss primarily emphasizes classification probabilities without imposing strong structural constraints on the feature space. Island loss, while introducing inter-class distance penalties, only enhances separation at the level of class centers. The standard ArcFace loss only explicitly constrains the angle between a sample’s features and its true class weight, as well as the angle between these features and other class weights. However, the angles between different class feature vectors are not directly constrained. Therefore, the performance improvement achieved by these three loss functions is considerably more limited compared to that of the proposed approach.

In summary, the overall training results demonstrate that the proposed approach consistently achieves superior performance throughout the training process, particularly during the later stages of convergence, exhibiting stronger discriminative capability and significantly outperforming the other three loss functions.

**Figure 9:**
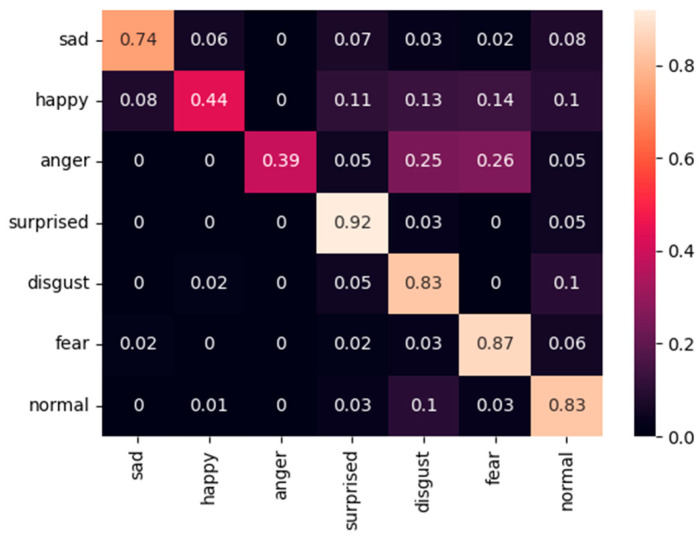
Confusion matric of our method on the FER2013 Public dataset.

**Figure 10:**
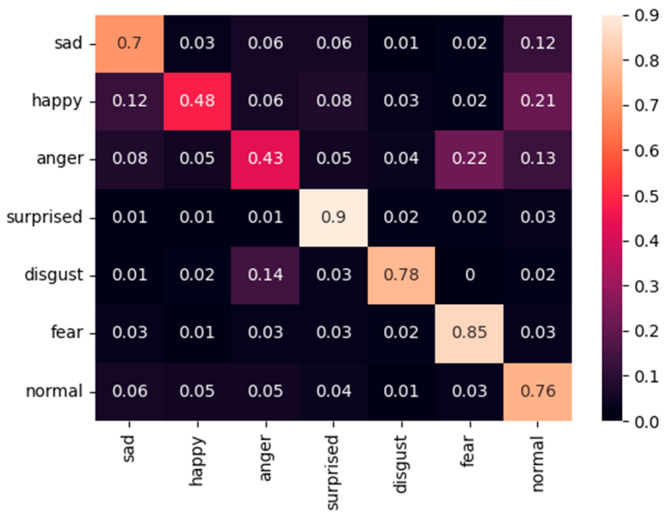
Confusion matric of standard ArcFace loss on the FER2013 Public dataset.

**Figure 11:**
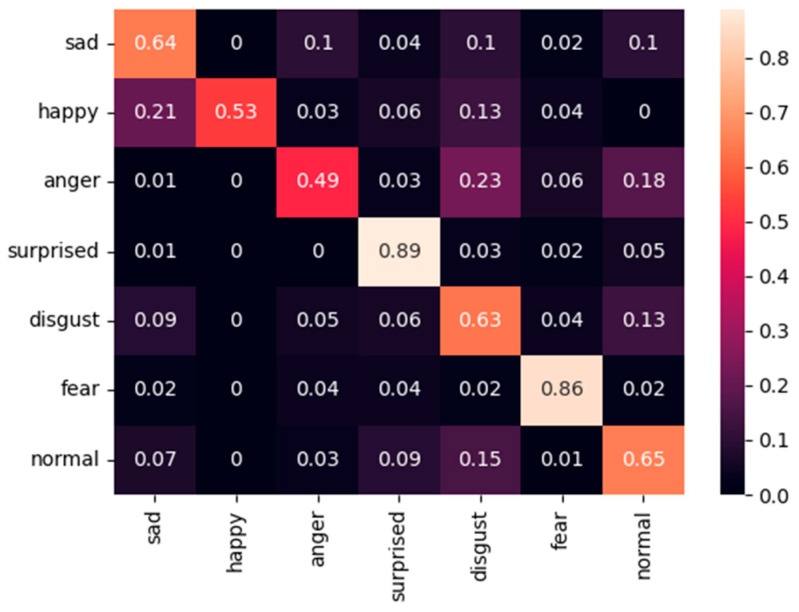
Confusion matric of Softmax-loss on the FER2013 Public dataset.

Figure 9, Figure 10 and Figure 11 present the confusion matrices obtained on the FER2013 Public test set using the proposed fixed-weight ArcFace loss, standard ArcFace loss, and Softmax loss. Compared with Softmax, which exhibits substantial confusion between visually similar categories such as sad–normal and anger–disgust, and standard ArcFace, which provides only partial improvement, the proposed method establishes clearer and more structured decision boundaries across all classes. For example, the accuracy of the “sad” category improves to 0.74 (Softmax: 0.64; ArcFace: 0.70). This improvement is accompanied by a marked reduction in misclassifications from “sad” to “normal.” The minority class “disgust” exhibits the most pronounced improvement, reaching 0.83 and significantly outperforming both Softmax (0.63) and ArcFace (0.78). The accuracy of the “fear” category improves to 0.87, further reducing confusion with the “surprised” class. The majority classes also benefit, as reflected in the improved performance of the “normal” category (0.83 vs. 0.65 for Softmax and 0.76 for ArcFace).

**Figure 12:**
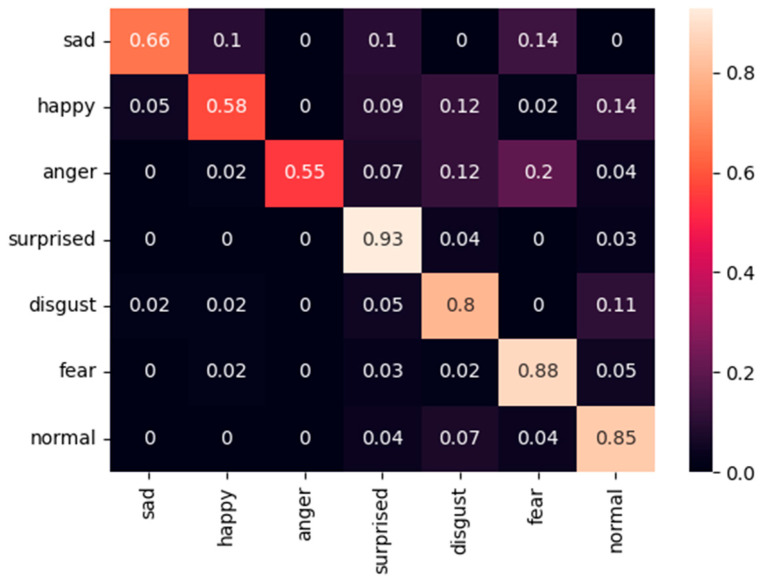
Confusion matric of our method on the RAF-DB dataset.

The proposed method exhibits comparable performance on the RAF-DB dataset, demonstrating consistent behavior across datasets. Figure 12 presents the corresponding confusion matrix, where the “surprised,” “fear,” and “normal” categories achieve accuracies of 0.93, 0.88, and 0.85, respectively. For more challenging expressions such as “sad,” “happy,” “anger,” and “disgust,” the model maintains strong recognition capability, achieving accuracies of 0.66, 0.58, 0.55, and 0.80, respectively. These results highlight the strong generalization capability of the proposed approach across different facial expression datasets, effectively enhancing inter-class separability and consistently capturing discriminative cues for both dominant and subtle emotional categories.

Overall, the proposed fixed-weight angular constraint significantly enhances inter-class separability and achieves notable gains for minority and highly confusable categories, demonstrating clear advantages over related approaches.

## 4. Conclusions

ArcFace loss offers several key advantages, including high computational efficiency, ease of implementation, and operational simplicity. It has attained state-of-the-art performance in numerous facial recognition benchmarks and exhibits strong adaptability, effectively handling large-scale image data while remaining compatible with video datasets. Furthermore, it seamlessly integrates into deep learning frameworks based on computational graphs.

In this study, the authors introduce a weight-constrained ArcFace loss function into the transformer framework, in which the improved loss function applies a weight constraint to the fully connected layer within the ArcFace formulation. This modification effectively reduces intra-class variations and enlarges inter-class separation, thereby significantly enhancing the recognition accuracy of visually similar categories, particularly in scenarios involving small facial expression datasets. Notably, the proposed approach achieves superior computational efficiency, reducing training time per epoch by approximately 1 s compared to traditional Softmax loss, 2–3 s compared to Island loss, and 0.5 s relative to standard ArcFace loss. The proposed approach exhibits simplicity, ease of implementation, and high computational efficiency.

However, as the number of expression categories increases, these methods may risk overwhelming the available weight space. Addressing this challenge through dynamic weight allocation or alternative optimization strategies represents a promising direction for future research.

## Figures and Tables

**Table 1:** Symbol and definition glossary for angular constraint and loss functions in facial expression recognition.

Symbol	Definition
$L_{1}$	Softmax-loss
$L_{2}$	Cosine-Softmax Loss
$L_{3}$	ArcFace Loss with angular margin
$L_{4}$	Inter-class angular constraint loss
L	Total loss function
$x_{i}$	Feature vector of the i-th sample
$W_{j}$	Weight vector of the j-th class
$b_{j}$	Bias term of the j-th class
$y_{i}$	Ground-truth class label of the i-th sample
*N*	Total number of training samples
*n*	Total number of classes
$\theta_{j}$	Angle between feature vector $x_{i}$ and weight vector $W_{j}$ of the j-th class
$\cos\theta_{j}$	Cosine similarity between feature vector $x_{i}$ and weight vector $W_{j}$ of the j-th class
*s*	Feature scaling factor
*m*	Angular margin in ArcFace Loss
*ω*	Weight coefficient of inter-class angular constraint loss $L_{4}$
*φ*	Weight coefficient of the inter-class angular constraint term in total loss
$W_{i}^{T}\cdot W_{j}$	Dot product of weight vectors of the i-th and j-th classes
$\theta_{ij}$	Angle between weight vectors of the i-th and j-th classes (i≠j)

**Table 2:** A comparative performance analysis of three models employing four distinct loss functions on the RAF-DB dataset, with the best results in bold.

	Model	Transformer	ResNet18	VGG19
Method				
Softmax-loss [9,10,11,12]		71.382	82.366	80.963
Island-loss [14]		71.089	83.472	81.672
ArcFace-loss [19]		71.683	84.126	82.040
**Our method**		**72.197**	**84.485**	**82.562**

**Table 3:** A comparative analysis of the performance of different loss functions for ResNet18 on the FER2013 dataset, with the best results displayed in bold.

	Test Set	Private	Public
Method			
Softmax-loss [9,10,11,12]		68.877	70.605
Island-loss [14]		69.260	71.684
ArcFace-loss [19]		69.685	70.828
**Our method**		**70.828**	**71.942**

**Table 4:** A comparative analysis of the average training speed per epoch across different loss functions on Transformer models using the RAF-DB dataset, with the best results displayed in bold.

Method	Average Training Time Per Round (S)
Softmax-loss [9,10,11,12]	37.28
Island-loss [14]	39.62
ArcFace-loss [19]	36.84
**Our method**	**36.50**

## Data Availability

The datasets used during the current study are the FER2013 dataset and the RAF-DB dataset, which are available online at https://www.kaggle.com/c/challenges-in-representation-learning-facial-expression-recognition-challenge/data (accessed on 20 October 2025), and http://www.whdeng.cn/RAF/model1.html (accessed on 20 October 2025), respectively.
